# Metabolomics analysis identifies glutamic acid and cystine imbalances in COVID-19 patients without comorbid conditions. Implications on redox homeostasis and COVID-19 pathophysiology

**DOI:** 10.1371/journal.pone.0274910

**Published:** 2022-09-20

**Authors:** José C. Páez-Franco, José L. Maravillas-Montero, Nancy R. Mejía-Domínguez, Jiram Torres-Ruiz, Karla M. Tamez-Torres, Alfredo Pérez-Fragoso, Juan Manuel Germán-Acacio, Alfredo Ponce-de-León, Diana Gómez-Martín, Alfredo Ulloa-Aguirre

**Affiliations:** 1 Red de Apoyo a la Investigación (RAI), Universidad Nacional Autónoma de México e Instituto Nacional de Ciencias Médicas y Nutrición Salvador Zubirán, Mexico City, Mexico; 2 Emergency Medicine Department, Instituto Nacional de Ciencias Médicas y Nutrición Salvador Zubirán, Mexico City, Mexico; 3 Department of Immunology and Rheumatology, Instituto Nacional de Ciencias Médicas y Nutrición Salvador Zubirán, Mexico City, Mexico; 4 Department of Infectology and Microbiology, Instituto Nacional de Ciencias Médicas y Nutrición Salvador Zubirán, Mexico City, Mexico; University of Florida, UNITED STATES

## Abstract

It is well known that the presence of comorbidities and age-related health issues may hide biochemical and metabolic features triggered by SARS-CoV-2 infection and other diseases associated to hypoxia, as they are by themselves chronic inflammatory conditions that may potentially disturb metabolic homeostasis and thereby negatively impact on COVID-19 progression. To unveil the metabolic abnormalities inherent to hypoxemia caused by COVID-19, we here applied gas chromatography coupled to mass spectrometry to analyze the main metabolic changes exhibited by a population of male patients less than 50 years of age with mild/moderate and severe COVID-19 without pre-existing comorbidities known to predispose to life-threatening complications from this infection. Several differences in serum levels of particular metabolites between normal controls and patients with COVID-19 as well as between mild/moderate and severe COVID-19 were identified. These included increased glutamic acid and reduced glutamine, cystine, threonic acid, and proline levels. In particular, using the entire metabolomic fingerprint obtained, we observed that glutamine/glutamate metabolism was associated with disease severity as patients in the severe COVID-19 group presented the lowest and higher serum levels of these amino acids, respectively. These data highlight the hypoxia-derived metabolic alterations provoked by SARS-CoV-2 infection in the absence of pre-existing co-morbidities as well as the value of amino acid metabolism in determining reactive oxygen species recycling pathways, which when impaired may lead to increased oxidation of proteins and cell damage. They also provide insights on new supportive therapies for COVID-19 and other disorders that involve altered redox homeostasis and lower oxygen levels that may lead to better outcomes of disease severity.

## Introduction

Efforts to unravel novel therapies to treat COVID-19 and post-COVID-19 manifestations are crucial due to the emergence of novel SARS-CoV-2 variants with augmented infectivity and/or pathogenicity. Clinical tests combining integral snapshots from the metabolic status of patients with COVID-19, have provided important information on the metabolic abnormalities provoked by SARS-CoV-2 infection [1–3], which may be useful for the design of new therapeutic approaches to counteract the negative effects of COVID-19 on the metabolic homeostasis. Alterations in the latter include the harmful effects of hypoxia and excessive reactive oxygen species (ROS) production that lead to severe cellular damage [4,5].

It is well known that decreased oxygen supply, incremented glycolysis, and activation of immune responses promote an altered redox state in COVID-19 and several malignancies [6–8]. These metabolic changes must be accompanied by a physiological response to support redox homeostasis. Synthesis, recycling, and/or supplementation of molecules with antioxidant activity [such as glutathione (GSH) or ascorbic acid] are essential to achieve ROS clearance [9], and in fact, several studies have shown that glutamine, GSH, and proline metabolism have a direct role on altered glutamic acid and cystine levels [10–13], which are amino acids that influence the synthesis of GSH, a molecule with antioxidant activity that has been recently revisited in the context of COVID-19 pathogenesis [4,14].

Several clinical studies focused on specific therapeutic interventions that increase the antioxidant capacity of COVID-19 patients have shown variable outcomes [15–18]. For example, administration of combined metabolic activators (e.g. glutathione and NAD+ precursors) or N-acetyl-cysteine (NAC) to a limited number of subjects, reduced the risk of mechanical ventilation and mortality as well as the plasma levels of metabolites associated with inflammation and inflammatory cytokines [15,16,18]. In contrast, administration of high dose zinc gluconate and/or ascorbic acid, which may positively impact on the immune response to infection, resulted in marginal effects on the duration of symptoms in mild to moderate COVID-19 [17]. On the other hand, it is well-known that the presence of comorbidities (*e*.*g*. metabolic syndrome, obesity, diabetes, and hypertension, among others) and age-related issues may hide important biochemical and metabolic characteristics derived from SARS-CoV-2 infection [19,20], as they may predispose to or are, *per se*, chronic inflammatory conditions that may alter the metabolic equilibrium as part of their pathophysiology and thereby significantly impact COVID-19 progression.

In the present study, we employed an untargeted metabolomic approximation to explore the main metabolic changes exhibited by a population of male patients less than 50 years of age with COVID-19, without pre-existing comorbidities (in particular, metabolic and cardiovascular diseases) that may convey greater risk of life-threatening complications from this infection and introduce confounding variables to our study findings [21–26]. In addition, we focused only in men considering the particular role of male endocrine environment on COVID-19 severity, including death [27–29]. Further, the metabolomic profile in women varies significantly with the menstrual cycle, which also may introduce confounding variables that prevent an accurate estimation of the relationship between changes in metabolomic profile and disease severity [30,31]. Under these considerations, the purpose of this analysis was to unveil those metabolic abnormalities inherent to COVID-19 respiratory illness that characterize patients with mild/moderate and severe disease, emphasizing on the metabolic alterations provoked by hypoxia in the context of redox homeostasis. We identified increased levels of glutamic acid, and phenylalanine and reduced cystine, glutamine, proline, threonic acid, and cholesterol levels. Our results highlight the value of amino acid metabolism in ROS recycling pathways and provide insights for new supportive therapies that may lead to better outcomes in this potentially catastrophic disease.

## Methods

### Subjects

To minimize the influence of gender, age and/or comorbidities on the metabolomics analysis performed [21–26,30,31], serum samples from twenty-two male COVID-19 patients (16 mild/moderate and 6 severe disease) with a positive RT-PCR test for SARS-CoV-2 as well as from sixteen RT-PCR negative healthy male volunteers (non-professional healthcare workers at our institution), less than 50 years of age (range 19 to 49 years), with no major pre-existing health conditions (*e*.*g*. metabolic syndrome, obesity, type-2 diabetes mellitus, cardiovascular diseases and/or other chronic diseases known to be associated with a poor prognosis of SARS-CoV-2 infection, as assessed by clinical history, physical examination, and/or routine biochemical tests [32]) were selected for the present metabolomic analysis. All subjects attended a third level referral center in Mexico City (Instituto Nacional de Ciencias Médicas y Nutrición Salvador Zubirán) from March to October 2020. Sixteen (72.7%) donor patients were classified as mild/moderate on the basis of previously published criteria [33] (fever, upper respiratory infection symptoms, with or without pneumonia, O_2_ saturation at rest >93% and respiratory rate <30 breaths per minute) and followed-up at home, whereas six (27.2%) patients were classified as severe [either respiratory failure, O_2_ saturation <93%, respiratory rate >30 breaths per minute or PaFi (PaO2/FiO2) ≤300 mmHg] and received in-hospital treatment. In subjects that met the inclusion criteria, blood samples were collected from an antecubital vein and placed in tubes containing clot-activating gel (BD Vacutainer, Becton Dickinson, Mexico). Tubes were inverted five times and the mixed blood was allowed to clot for 30 minutes at room temperature, centrifuged at 1300 x g for 15 minutes, and the resulting serum was then collected with a sterile disposable Pasteur pipette, aliquoted in cryogenic vials, and stored at -80°C until GC/MS analysis. All included samples were drawn upon admission to the triage area at the emergency department for the patients or at the epidemiology unit for the controls, where hospital staff members attended for randomized nasopharyngeal PCR testing during the morning shift and in a fasting state (minimum 8 h). The study was approved by the institutional ethics and research committees of our institute (Ref. RAI3688/21/22/1). Written informed consent to participate in the study was obtained from all participants. All analytical methods were carried out in accordance with relevant and updated guidelines and regulations.

### Gas chromatography/mass spectrometry (GC/MS) analysis

Metabolomics analysis was performed by GC/MS as previously described, with minor modifications [34]. Briefly, 35 μL of serum were mixed with 150 μL of 1:3 chloroform-methanol and 5 μL of internal standard (tridecanoic acid, methyl tricosanoate, and 5α-cholestane, 0.18 mg/mL each) and vortexed for 2 min. The mix was incubated for 20 min at -20°C and then centrifuged at 16000 x g during 10 min at 4°C. The supernatant unique phase was collected, evaporated in a constant nitrogen flux, resuspended in 40 μL of methoxyamine in pyridine and incubated for 90 min at 37°C. Thereafter, 40 μL of N-methyltrifluoroacetamide (MBSTFA) plus 1% trimethylchlorosilane (TMCS) was added to the final solution and incubated for 30 min at 37°C. Finally, one microliter was injected (splitless) to a GC/MS system (Agilent 5977A/7890B, Santa Clara, CA, USA) with an autosampler (G4513A, Agilent) and run in a HP-5ms column (30 m × 250 μm × 0.25 μm; Agilent) with helium 99.9999% as a mobile phase and at 1 ml/min flow, 200°C inlet temperature, 200°C source temperature, and 250°C interface temperature. The running method consisted of 1 min hold at 60°C with an increased ramp of 10°C/min to 325°C, with a final held time of 10 min. Samples were injected aleatorily and a quality control sample (a mix of all the samples) was included every six samples to monitor changes in instrument acquisition and to ensure reproducibility. Only those peaks with less than 30% relative standard deviation (RSD) in quality control samples were included [35]. All reagents and solvents employed were pure analytical grade materials purchased from commercial sources.

### Chromatogram deconvolution

Raw GC/MS data was transformed using the Agilent Chemstation software (Agilent), and the chromatogram deconvolution and alignment were processed with the Mzmine 2 software [36] using the following parameters: retention time (RT) range, 5.5–27.5 min; m/z range, 50–500; m/z tolerance, 0.5; noise level, 1×10^3^; and peak duration range, 0.01–0.2 min. Only those peaks that appeared in 80% of all the chromatograms were included. Thereafter, the identification was achieved using the National Institute of Standards and Technology (NIST) spectral library. Only those metabolites with a matching value >70% were included in the final analysis. The raw data has been submitted to MetaboLights data base with the accession number MTBLS5011.

### Statistical analysis

To analyze for differences among control and patients groups, the U-Mann-Whitney test or Kruskal-Wallis test were applied using Graphpad Prism software 8.0 (GraphPad Software Inc., San Diego, CA) and adjusted by False Discovery Rate analysis. A probability value of *p*<0.05 was considered statistically significant. Spearman´s rank correlation analysis, heatmaps with hierarchical clustering, and fold change analysis were performed in Metaboanalyst 5.0 and R project software [37,38] using data with sum normalization. Changes in those metabolites with a fold change (FC) >1.5 and p-value <0.05 were considered significant. Principal component analysis (PCA) and partial least squares-discriminant analysis (PLS-DA) also were performed through Metaboanalyst 5.0 previous sum normalization, data log transformation, and mean centering. Data normalization through internal standard (tridecanoic acid) showed similar results. Metabolite set enrichment analysis was generated in Metaboanalyst 5.0, employing the KEGG human metabolic pathways database (.genome.jp/kegg/pathway.html#metabolism).

## Results

The main characteristics of the cohort studied are listed in Table 1. Further biochemical tests (shown in S1 Table) were performed in the patients included in the severe COVID-19 group. All patients in the mild/moderate group were followed-up through daily phone calls for 2 weeks since the time of COVID-19 diagnosis and none progressed to severe or critical forms of the disease. None of the patients included in mild/moderate or the severe groups progressed to a more advanced condition, and all were followed until their discharge from the hospital.

**Table 1:** Main demographic features of the patients included in the analysis.

Demographic features	Healthy n = 16	Mild/Moderate* n = 16	Severe n = 6	*p-*value
Age	34.9 ± 6.2	33.2 ± 9.4	40.8 ± 8.0	^1^N.S.
BMI	25.9 ± 2.9	25.8 ± 3.5	28.2 ± 1.5	^1^N.S.
Respiratory rate (bpm)	-	19.1 ± 2.6	24.8 ± 5.5	^2^<0.01
SO2 (%)	-	94.7 ± 1.5	86.7 ± 8.3	^2^<0.001

*p*-value for

^1^Kruskal–Wallis or

^2^U Mann-Whitney test.

* No patient in this group progressed to severe disease.

**Fig 1:**
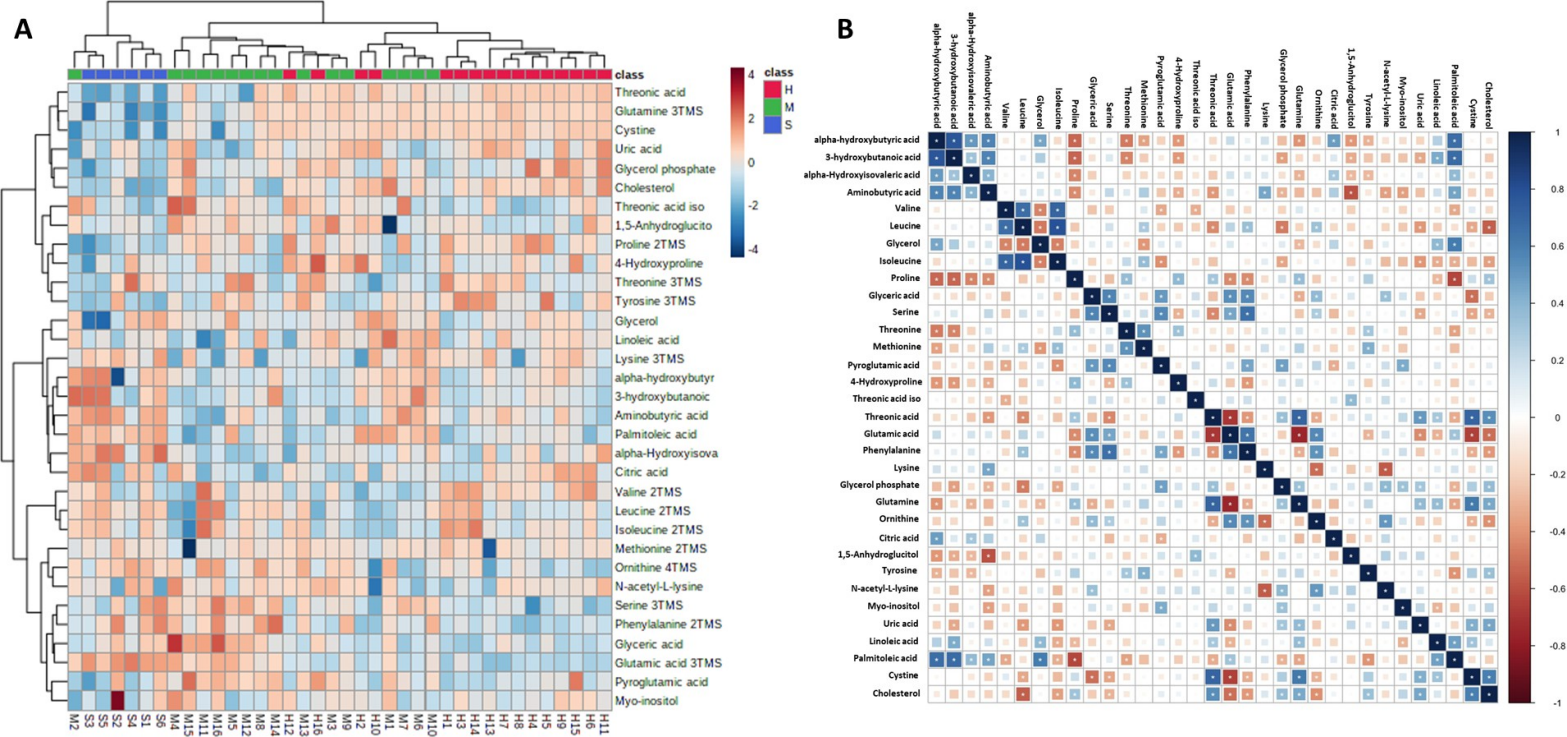
Untargeted serum metabolomics of COVID-19 patients. A. Heatmap and hierarchical clustering analysis of COVID-19 and control individuals with all the metabolites included in the analysis. Different intensities in red and blue colors in the heatmap and scale bar at the right side of the figure, denotes increased or reduced levels, respectively. B. Spearman correlation analysis of serum metabolites in COVID-19 and control groups. H, healthy controls, M, mild/moderate disease; S, severe disease. Blue numbers indicate a positive association. Red numbers indicate a negative association. Asterisks denote statistical significance with p-value < 0.05. The exact p-values of Spearman´s correlation analysis is depicted in S3 Table.

Our untargeted metabolomic approach consistently identified 33 different metabolites (S2 Table). To ensure that our technique was reproducible throughout the study, Principal Component Analysis (PCA) was applied to all samples; a well-defined QC cluster was noticed, indicating that our method was robust enough to analyze the cohort’s metabolomic profile (S1 Fig). Increased glutamic acid, and reduced glutamine, cystine, and threonic acid levels were detected in both the mild/moderate and severe COVID-19 patients, whereas proline levels significantly decreased only in the severe COVID-19 group. The majority of these changes were more marked in patients with severe disease. The statistical analysis for those metabolites that exhibited a FC>1.5 is shown in Table 2 and the complete set of all metabolites in S2 Table. To visualize these differences, heatmap and hierarchical clustering analyses were applied (Fig 1A). As shown, our patients segregated with good performance and according with disease severity.

**Table 2:** Metabolites with significant changes between the groups.

#	Metabolite–ID	HMDB–ID	Log2 Fold Change/ Fold Change	Kruskal Wallis ♣ *p*-adjusted value		
				H *vs* M	H *vs* S	M *vs* S
1	Proline	HMDB00162	H/S 0.93747 (FC 1.9152) M/S 0.59025 (FC 1.5055)	0.1115	↓ *** 0.0006	0.0901
2	Threonic acid	HMDB00943	H/S 3.6513 (FC 12.564) H/M 0.75051 (FC 1.6824) M/S 2.9008 (FC 7.4682)	0.0747	↓*** 0.0001	↓* 0.0481
3	Glutamic acid	HMDB00148	H/S -3.0884 (FC 0.11757) H/M -1.7131 (FC 0.30501) M/S -1.3754 (FC 0.38545)	↑** 0.0013	↑**** <0.0001	0.0919
4	Phenylalanine	HMDB00159	H/S -0.64267 (FC 0.64053)	↑* 0.0237	0.0816	>0.9999
5	Glutamine	HMDB00641	H/S 2.4319 (FC 5.3961) M/S 2.1116 (FC 4.3218)	0.2942	↓*** 0.0002	↓* 0.0148
6	Cystine	HMDB00192	H/S 2.6738 (FC 6.3812) H/M 0.61863 (FC 1.5354) M/S 2.0552 (FC 4.156)	↓ * 0.0196	↓ **** <0.0001	0.0928
7	Cholesterol	HMDB00067	H/S 0.85438 (FC 1.808)	↓ * 0.0248	↓ *** 0.0008	0.2654

*Kruskal Wallis-Dunn test. H, healthy controls; M, mild/moderate COVID-19; and S, severe COVID-19. ↑: increment; ↓: decrement.

To identify potential associations between the metabolites and disease severity, we performed Spearman correlation analysis, which revealed that glutamic acid correlated negatively with cystine, threonic acid, and glutamine (Fig 1B). Likewise, cystine correlated positively with threonic acid and glutamine. The family of branched chains amino acids (valine, leucine, and isoleucine) correlated positively among each other, albeit these changes did not reach statistical significance when analyzed by group severity (S2 Table).

**Fig 2:**
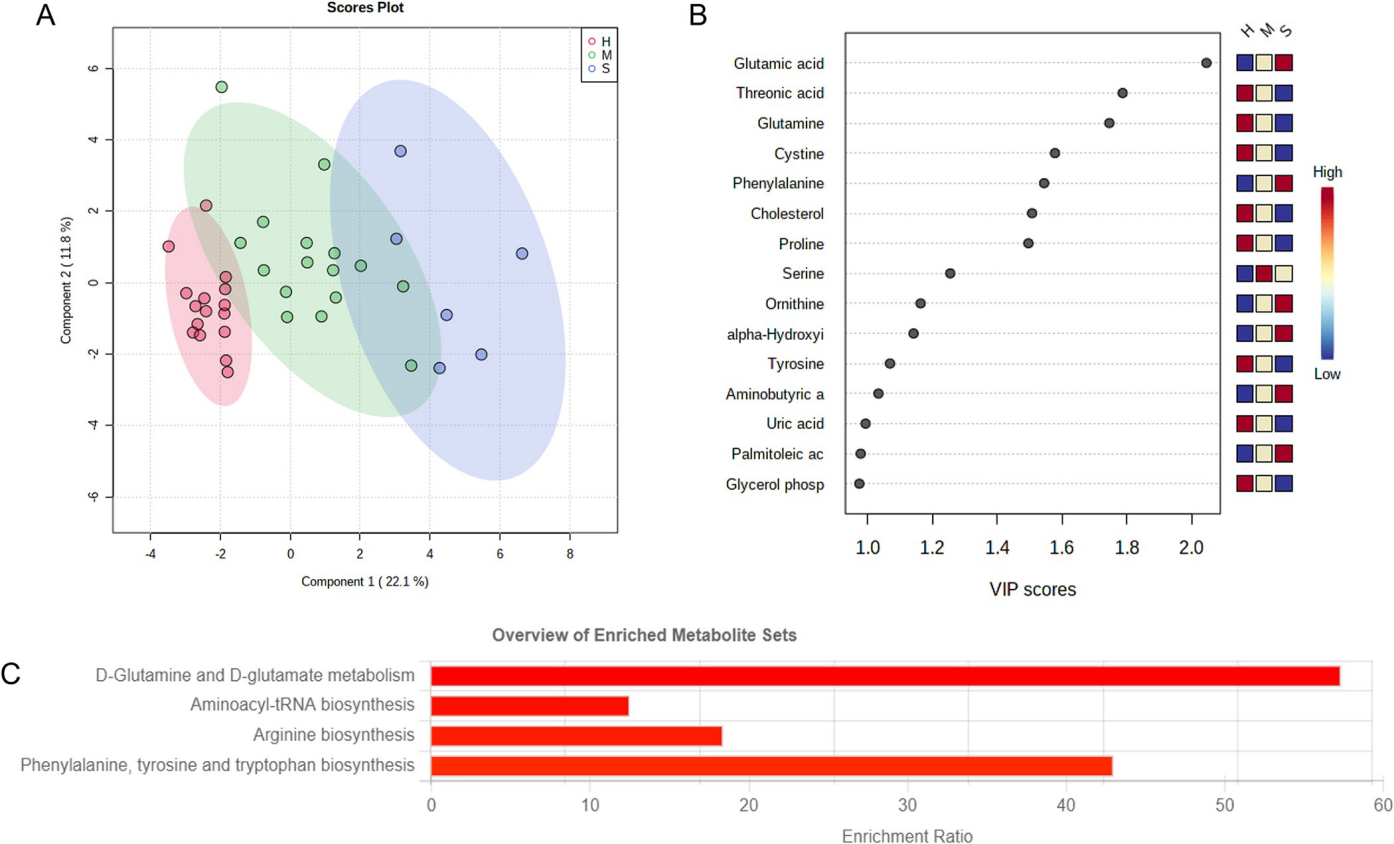
Partial least squares-discriminant analysis of COVID-19 patients. A. Score plot of PLS-DA analysis of healthy controls (H), mild/moderate (M), and severe (S) patients. B. Variable importance in the projection (VIP) values that better explain the differences between the groups; C. Enriched metabolite data sets (p<0.05) for the VIP metabolites involved in COVID-19 severity clustering.

To further discriminate between disease severity, PLS-DA was applied to the data, showing a good performance (Accuracy = R2 = 0.81, Q2 = 0.73, p<0.05) (Fig 2A). The variable importance in projection (VIP) scores are summarized in Fig 2B and corresponds to the metabolites that better discriminate the groups according to disease severity. Employing this set of metabolites, we implemented pathway enrichment analysis in Metaboanalyst 5.0 and observed statistical significance (p<0.05) and enrichment ratio of glutamine/glutamate metabolism (Fig 2C).

**Fig 3:**
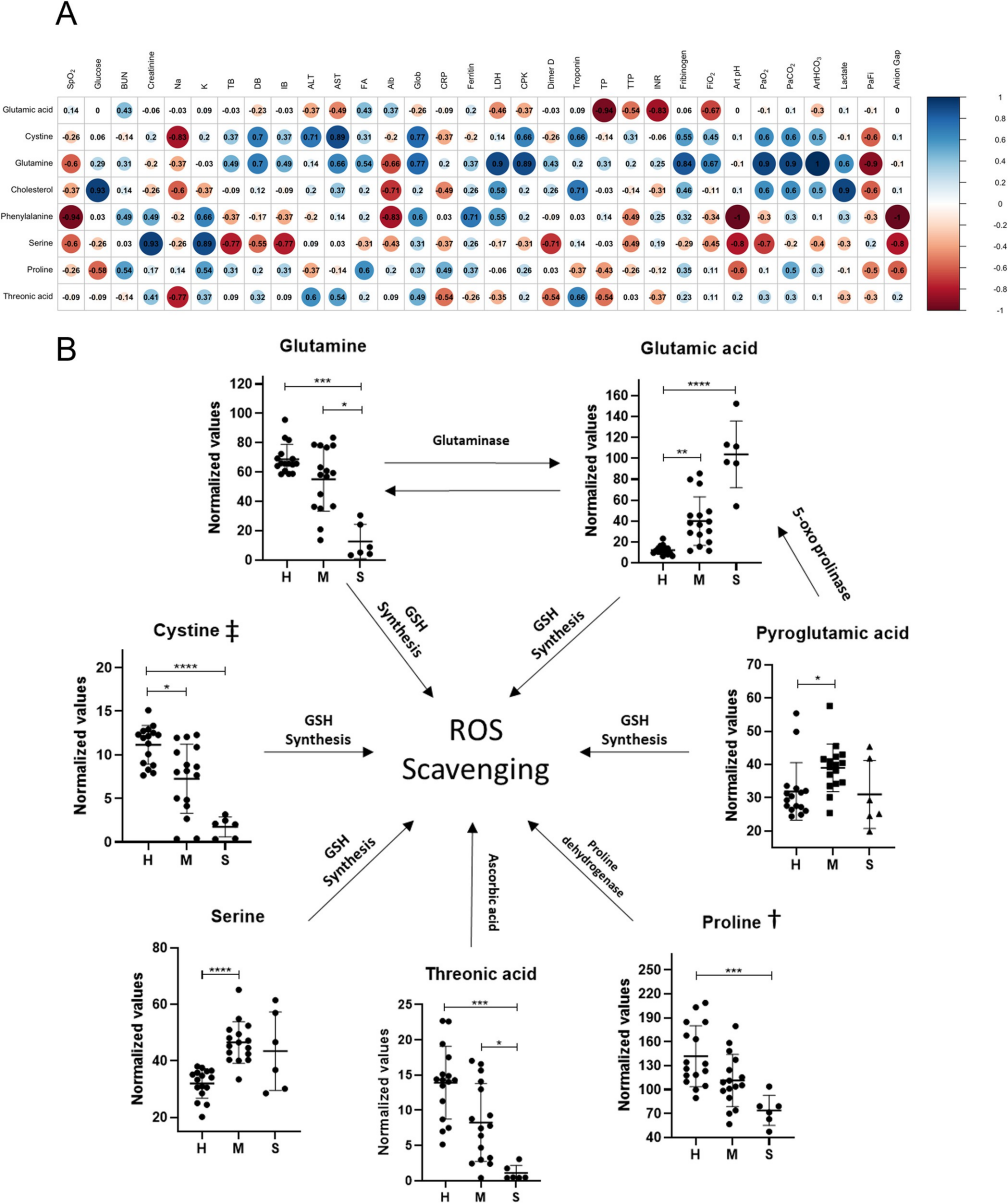
Correlation analysis of the metabolites and the clinical tests in the severe group. A. Spearman correlations analysis of metabolites and clinical tests. SpO2 = Oxygen saturation; LDH = Lactate dehydrogenase; Art pH = Arterial pH; PaO_2_ = Partial pressure of oxygen; PaCO_2_ = Partial pressure of CO_2_; Art HCO_3_ = Arterial bicarbonate; PaFi = PaO2/FiO2; TB = Total bilirubin; DB = Direct bilirubin; IB = Indirect bilirubin; ALT = Alanine aminotransferase; AST = Aspartate aminotransferase; AP = Alkaline phosphatase; Alb = Albumin; INR = International Normalized Ratio; CRP = C reactive protein; CPK = Creatine phosphokinase. Different intensities in blue and red numbers indicate a positive or negative association, respectively (see also the gradient scale at the right side of the figure). B. Proposed model for the main changes identified in our cohort. The graphs were constructed with normalized values. H, healthy controls; M, mild/moderate disease; S, severe disease. ‡Direct involvement of cystine over glutamic acid levels through xCT antiporting system. †Enzymatic involvement over glutamic levels through proline dehydrogenase activity. Statistical differences were calculated employing Kruskal-Wallis and Dunn tests. P*<0.05 **<0.01, ***<0.001, ****<0.0001.

Metabolite association (VIP>1.2) with the clinical features in the severe group is depicted in Fig 3A. Glutamine positively correlated with LDH, CPK, PCO_2_, and HCO_3_, and negatively with PaFi. On the other hand, glutamic acid showed negative associations with prothrombin time (PT), International Normalized Ratio (INR), and FiO_2_. Moreover, cystine and threonic acid showed a positive correlation with alanine aminotransferase (ALT) and aspartate aminotransferase (AST), both markers of hepatic function. Conversely, cystine and threonic acid showed negative associations with sodium levels. Phenylalanine correlated negatively with SpO_2_, albumin, pH, and anion gap markers. The main changes observed in several amino acids and other metabolites are shown in Fig 3B. Glutamine, cystine, threonic acid (a sugar acid derived from threose), and proline, were progressively lower in patients with mild/moderate and severe disease, whereas glutamic acid and serine were significantly higher as compared with the healthy controls; the latter amino acid, albeit elevated in both COVID-19 groups, did not reach statistical significance in the severe group probably due to the reduced number of patients included in this group. The impact of the main changes observed in our cohort on ROS scavenging is also depicted in Fig 3B.

## Discussion

The main complications derived from SARS-CoV-2 infection are related to systemic oxygen shortage due to lung tissue damage as a result of exacerbated immune activation and virus replication, which lead to metabolic changes that prepare target organs and tissues to reduced oxygen supply [39]. Leaving aside the metabolic reshaping provoked by the virus on infected cells [40,41], it has been proposed that critical metabolic changes occur in endothelial and immune cells in the early stages of lung damage [40]. Moreover, if oxygen levels do not recover, the decrement in oxygen supply may lead to further damage to critical organs such as brain, kidneys, and liver [42,43], as observed in critical COVID-19 patients.

In the present study, we identified several differences in serum levels of particular metabolites among healthy controls and patients with mild/moderate or severe forms of COVID-19, which presumably were not related to any co-morbidity associated to a negative prognosis [32]. Immune-activated and oxygen-deprived cells show metabolic signatures associated with the increase in glycolytic pathways and glutamine metabolism that feed energetic and biosynthetic processes [44–47]. In this vein and in line with other studies [48–53] we observed decreased serum glutamine and high glutamic acid levels in patients with severe COVID-19. Further, applying pathway enrichment analysis, we detected statistical significance and enrichment ratio of glutamine/glutamate metabolism. Here is important to emphasize in that only 33 metabolites were identified from the sera by the GC/MS method employed of which only 15 were used in the enrichment pathway analysis. Therefore, it should not be surprising that the D-glutamine and D-glutamate metabolic pathways resulted highly enriched by this analysis. Although the results of this analysis fitted very well with the analytic data (Fig 3B), further studies including a larger number of metabolites are needed to confirm the results from the pathway enrichment analysis performed. Although different metabolomics analyses of samples from COVID-19 patients have detected increased levels of glutamate, this change still is poorly understood and underestimated despite that this amino acid acts as a neurotransmitter that when unbalanced, may lead to excitotoxicity and neurological damage, both observed in COVID-19 pathophysiology [54]. In fact, it is known that hypoxic-ischemic conditions may promote cognitive alterations [55,56] that to some extent, could resemble COVID-19 and long COVID-19 neurological disabilities [57,58]. The importance of glutamine metabolism in patients and experimental animals with COVID-19 is well documented [10,59,60] and in fact a recent analysis concluded that glutamine supplementation reduced time of hospitalization and the necessity of intensive care of these patients [61]. Further, the markedly decreased plasma levels of glutamine in patients with severe inflammatory condition due to COVID-19, rapidly reverted in those with a favorable outcome [62]. Glutamine-derived glutamate can feed several pathways like the Krebs cycle, gluconeogenesis, and GSH synthesis (Fig 3B), and its role in hypoxia is critical as it maintains energy and redox homeostasis in stress conditions [12]. In this regard, it has been observed that GSH levels are decreased in COVID-19 [4] and that supplementation with substrates that regenerate GSH (like N-acetylcysteine) may be favorable to reduce the risk for mechanical ventilation [15].

The endogenous source of glutamic acid derives from glutaminase activity, an enzyme that produces glutamic acid from glutamine in a process called glutaminolysis. A different source of glutamic acid is the gamma-glutamyl cycle, which is the synthetic pathway of GSH, an important tripeptide with antioxidant activity. Dysregulation of this cycle promotes the increment of glutamic and pyroglutamic acid [63]. Individuals with glutathione synthetase deficiency or paracetamol overdose show increased pyroglutamic acid levels, which when severe, may lead to metabolic acidosis and liver failure [63,64]. In the present study, we detected a slight increase (<1.5 fold change, p<0.05) in pyroglutamic acid levels in mild/moderate COVID-19 patients, a change that has been identified in other COVID-19 cohorts [59,65,66]. In our study, pyroglutamic changes were apparently unrelated to altered hepatic function, as disclosed by biochemical tests (S1 Table). Pyroglutamic acid modest increment in mild/moderate COVID-19 patients have been associated with alterations in GSH synthesis provoked by decreased supply of substrates like ATP, glycine or cysteine in environments with ROS excess [67]. In this regard, we observed decreased cystine levels, which is the predominant extracellular form of cysteine.

Cysteine or cystine imbalance has been reported in other COVID-19 cohorts with variable changes according to disease severity, immune activity and presence of comorbidities [1,33,51,68–70]. In fact, cystine levels are critical to contend with ROS in COVID-19 as well as in several malignancies [1]. In the present study, reduced cystine levels correlated positively with ALT and AST levels in patients with severe COVID-19. In this vein, a potential hepatic involvement in cysteine and cystine turnover in COVID-19 seems plausible as this organ, which is frequently compromised in the severe form of this disease, is the main place of GSH synthesis [71,72]. Although cystine correlates negatively with glutamic acid, the counterion implied in the xCT antiporting system of cystine [73], the influence of this system on increased serum glutamic acid levels is not clear yet. Recently, a glutamate/cystine antiporter system that favors GSH synthesis in ROS was described in malignant lung cells [74] and it has been found that the transporter xCT is increased in monocytes from COVID-19 patients [51], suggesting that cystine has probably a role in immunity [75]. Interestingly, in an example of metabolic flexibility, cystine and glutamic acid levels influence glutamine anaplerotic usage in malignant cells through the xCT transporter [76]. Although the occurrence of this effect during hypoxia still remains unexplored, it is known that serum glutamine levels and its anaplerotic reactions are essential to reduce SARS-Cov-2 pathogenicity [10].

On the other hand, it was previously shown that reduced cystine levels correlated positively with threonic acid, a catabolite of ascorbic acid, the primary antioxidant obtained from food [77]. This relationship indicates that redox homeostasis was probably altered in our cohort. In this regard, it has been observed that ascorbic acid levels tend to be lower in severe COVID-19 patients [78]. Although our metabolomic analysis suggests that ascorbate supply might surpass redox imbalances, its exogenous supplementation has not shown any benefit, at least in ambulatory patients with COVID-19 [17].

In agreement with findings from other studies [1,2,79–81], we detected decreased proline levels in those patients with severe COVID-19 studies. In this vein, it has been shown that decreased proline levels are associated with an unfavorable prognosis in patients with the severe form of this disease [80,82]. Further, in a genomewide association study of severe COVID-19 a locus spanning six genes showing cross replicating associations with disease severity was identified and, interestingly, one of these genes codes the transporter SIT-1, a protein that interacts with angiotensin converting enzyme 2 (ACE), the SARS-CoV-2 cell receptor [83] and that is directly involved in the transport of proline, an amino acid associated to protein folding. These data suggest that proline metabolism could be related with a stress response in COVID-19, more specifically to an exaggerated unfolded protein response in helper T-lymphocytes and macrophages, thereby contributing to intracellular hypoxia and a pathogenic cytokine response. In this regard, *in vitro* studies with cardiomyocytes expressing higher levels of proline dehydrogenase revealed that enhanced proline metabolism reduced apoptosis and decreased hypoxia derived from ROS [84]. In this same line of evidence, it has been shown that the activity of proline dehydrogenase increases the protection to ROS in malignant melanoma cells and also promotes release of glutamic acid and alpha-ketoglutarate as reaction byproducts [85]. This reaction can feed anaplerotic reactions in stress conditions and could partially explain some of the increased glutamic acid levels in COVID-19 [11].

Finally, we observed a modest, but significant increase (FC<1.5, p-value<0.05) in levels of serine in patients with mild/moderate and severe COVID-19, albeit to a lower extent in the latter group. Altered levels of serine associated with variable outcomes have been documented in other metabolomic analyses [68,86,87]. In this regard, it was shown that serine supplementation decreased ROS damage in mice with oxidative stress through direct involvement in GSH synthesis [88]; the property of promoting glycolysis and GSH production is apparently unique to this amino acid [89].

We recognize that the number of patients included in this metabolomic analysis was limited, given that the study design did not consider subjects with co-morbidities associated with high COVID-19 mortality and which may potentially confound the metabolomic analysis [21–26,31]. Frequently associated COVID-19 co-morbidities including obesity, diabetes mellitus and cardiovascular diseases, all comprise ~80% of the patients attended in our third level health center, which made quite difficult to recruit for the present metabolomics study patients free of co-morbidities. In this vein, our findings are comparable with other studies including larger numbers of patients and presence of comorbidities in which altered levels of branched-chain amino acids, glutamine, glutamate, and proline also were identified, suggesting that neither differences in patient number or abscence of comorbidities influenced on the general metabolic reshaping provoked by SARS-CoV2 infection [1,79,81,90–93]. Another limitation is that we did not include additional controls with other viral pneumonias in which hypoxia and respiratory failure may lead to altered metabolomics, particularly when associated to hypoxic liver injury [94,95]. Nevertheless, in this vein it should be emphasized that during the COVID-19 pandemic, the incidence of other opportunistic and/or viral pneumonias dramatically decreased in several regions, including ours [96–99] most probably due to the mandatory measures imposed for COVID-19 prevention [99]. Finally, our metabolomics study was limited to males as it is well known that the metabolomic profile in premenopausal women varies with the menstrual cycle, particularly during the luteal phase. In this vein, studies employing GC/MS and nuclear magnetic resonance detected significant changes in a number of amino acids and derivatives, biogenic amines, and lipid species during the luteal phase of the menstrual cycle associated with the increased levels of serum progesterone that characterize this cycle phase [30,31]. Thus, we decided to include only males in our study given that for a mixed gender cohort it would have been necessary to include a relatively large number of women during at least the early, late follicular/preovulatory, and luteal phases of the menstrual cycle in order to statistically analyze the impact of each cycle phase on the metabolic changes exhibited by control and COVID-19 women, at least by multivariate analysis.

In conclusion, this study documents the existence of altered levels of several metabolites in COVID-19 patients in the absence of frequently associated co-morbidities. Assessing the magnitude of these alterations in both mild/moderate and severe COVID-19 patients is important as they seem to play a pivotal role in immune response and ROS scavenging. Our data emphasize the potential importance of amino acid supplementation in COVID-19 patients to prevent not only further progression of the disease but also probably the development of the chronic form of this infection.

## Supporting information

S1 Fig Principal component analysis of all patients included in the analysis. Pooled samples (blue intense) were used as a quality control and shows a compact well-defined cluster, ensuring a good reproducibility of our GC/MS analysis.
(DOCX)

S1 Table Entire set of clinical test performed in the severe group. (DOCX)

S2 Table List of metabolites included in our GC/MS analysis. (DOCX)

S3 Table p-values of Spearman´s correlation analysis in Fig 1B. (DOCX)
