# Supplementary material for: Metabolomics analysis identifies glutamic acid and cystine imbalances in COVID-19 patients without comorbid conditions. Implications on redox homeostasis and COVID-19 pathophysiology
Source: PLoS One. 2022 Sep 20;17(9):e0274910. doi: 10.1371/journal.pone.0274910 (PMC9488784; doi:10.1371/journal.pone.0274910)
Supplement: S1 Fig — Pooled samples (blue intense) were used as a quality control and shows a compact well-defined cluster, ensuring a good reproducibility of our GC/MS analysis. (DOCX) [file pone.0274910.s001.docx]

**Metabolomics Analysis Identifies Glutamic acid and Cystine imbalances in COVID-19 Patients Without Comorbid Conditions. Implications on Redox Homeostasis and COVID-19 Pathophysiology**

José C. Páez-Franco, José L. Maravillas-Montero, Nancy R. Mejía-Domínguez, Jiram Torres-Ruiz, Karla M. Tamez-Torres, Alfredo Pérez-Fragoso^3^ Juan Manuel Germán-Acacio^1^, Alfredo Ponce-de-León^4^, Diana Gómez-Martín^3^, and Alfredo Ulloa-Aguirre

**Supplementary information, S1 Figure**


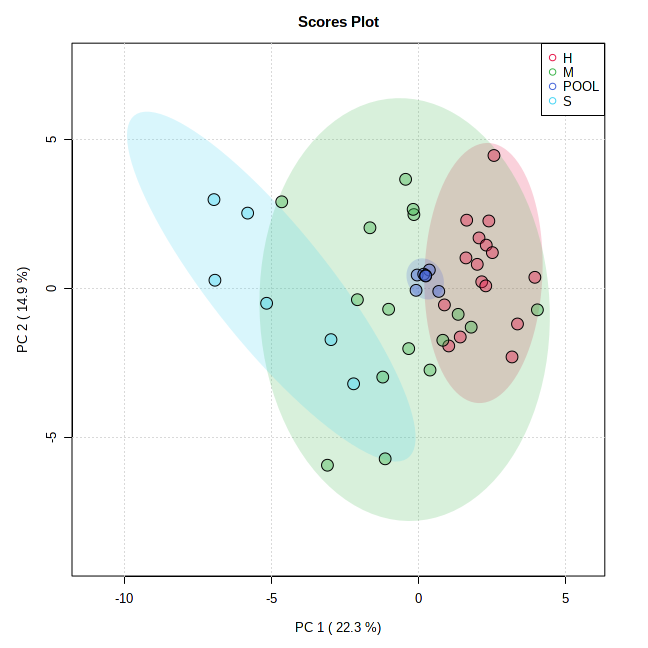


S1 Figure. Principal component analysis of all patients included in the analysis. Pooled samples (blue intense) were used as a QC and shows a compact well-defined cluster, ensuring a good reproducibility of our GC/MS analysis.
