# Supplementary material for: Metabolomics analysis identifies glutamic acid and cystine imbalances in COVID-19 patients without comorbid conditions. Implications on redox homeostasis and COVID-19 pathophysiology
Source: PLoS One. 2022 Sep 20;17(9):e0274910. doi: 10.1371/journal.pone.0274910 (PMC9488784; doi:10.1371/journal.pone.0274910)
Supplement: S1 Table — (DOCX) [file pone.0274910.s002.docx]

**Metabolomics Analysis Identifies Glutamic acid and Cystine imbalances in COVID-19 Patients Without Comorbid Conditions. Implications on Redox Homeostasis and COVID-19 Pathophysiology**

José C. Páez-Franco, José L. Maravillas-Montero, Nancy R. Mejía-Domínguez, Jiram Torres-Ruiz, Karla M. Tamez-Torres, Alfredo Pérez-Fragoso^3^ Juan Manuel Germán-Acacio^1^, Alfredo Ponce-de-León^4^, Diana Gómez-Martín^3^, and Alfredo Ulloa-Aguirre

**Supplementary information, S1 Table**

S1 Table. Clinical and laboratory data from patients in the severe Covid-19 group.

| Clinical test / Patient | 1 | 2 | 3 | 4 | 5 | 6 |
| --- | --- | --- | --- | --- | --- | --- |
| Body mass index (kg/m^2^) | 29.2 | 28.1 | 29.4 | 25.4 | 29.6 | 28.8 |
| Mean Arterial pressure (mmHg) | 88 | 96.6 | 116 | 86.6 | 78 | 94.6 |
| Heart rate (beats per minute) | 125 | 115 | 100 | 102 | 105 | 120 |
| Respiratory rate (breaths per minute) | 26 | 35 | 24 | 20 | 24 | 20 |
| Oxygen saturation (SpO2, %) | 89 | 70 | 92 | 91 | 90 | 88 |
| Glucose (mg/dL) | 89 | 122 | 105 | 96 | 105 | 102 |
| Blood Urea Nitrogen (mg/dL) | 10.7 | 31.7 | 6 | 14.1 | 11.9 | 12 |
| Creatinine (mg/dL) | 1.13 | 0.95 | 0.95 | 0.8 | 0.78 | 1.09 |
| Sodium (mmol/L) | 135 | 132 | 136 | 140 | 138 | 139 |
| Potassium (mmol/L) | 5.01 | 4.16 | 3.87 | 3.92 | 3.76 | 4.23 |
| Total bilirubin (mg/dL) | 0.68 | 0.61 | 0.57 | 0.85 | 0.97 | 0.49 |
| Direct bilirubin (mg/dL) | 0.2 | 0.19 | 0.16 | 0.19 | 0.26 | 0.15 |
| Indirect bilirubin (mg/dL) | 0.48 | 0.42 | 0.41 | 0.66 | 0.71 | 0.34 |
| Alanine aminotransferase (U/L) | 59.5 | 39.2 | 51.5 | 17.2 | 48 | 36 |
| Aspartate aminotransferase (U/L) | 60.1 | 54.6 | 31.5 | 16 | 60 | 31 |
| Alkaline phosphatase (U/L) | 56 | 140 | 46 | 92 | 78 | 52 |
| Albumin (g/dL) | 4 | 3.16 | 4.5 | 5.2 | 3.6 | 3.54 |
| Globulins (g/dL) | 3.54 | 3.47 | 2.5 | 2.7 | 3.3 | 2.94 |
| C reactive protein (mg/dL) | 13.76 | 9.62 | 2.28 | 27.85 | 17.29 | 15.7 |
| Ferritin (ng/dL) | 732 | 1835 | 394 | 911 | 807 | 1242 |
| Lactate dehydrogenase (U/L) | 395 | 771 | 225 | 395 | 883 | 422 |
| Creatine phosphokinase (U/L) | 751 | 550 | 48 | 113 | 766 | 76 |
| D Dimer (ng/mL) | 371 | 826 | 374 | 1772 | 6375 | 496 |
| Troponin I (pg/mL) | 6.1 | 165 | 28 | 3.1 | 5.1 | 3.6 |
| TP | 13.2 | 10.6 | 12.4 | 12.2 | 14.2 | 13.8 |
| TTP | 32.7 | 24.9 | 32.5 | 32.2 | 35 | 29.9 |
| International Normalized Ratio | 1.2 | 0.9 | 1.1 | 1.1 | 1.2 | 1.2 |
| Fibrinogen (mg/dL) | 687 | 789 | 415 | 687 | 705 | 600 |
| FiO2 | 30 | 21 | 21 |  | 40 | 21 |
| pH | 7.48 | 7.47 | 7.56 |  | 7.52 | 7.46 |
| PO2 | 76.4 | 83.2 | 64.7 |  | 92 | 55.9 |
| PaCO2 (mmHg) | 26.1 | 31.4 | 18 |  | 26.9 | 25.2 |
| HCO3 (mmHg) | 19.4 | 22.7 | 16.1 |  | 23 | 17.6 |
| Lactate (mmol/L) | 1 | 2.1 | 1.3 |  | 1.6 | 1.4 |
| PaFi | 255 | 139 | 308 |  | 230 | 266 |
| Anion Gap (mmol/L) | 15.2 | 14.2 | 15.8 |  | 15.4 | 12.9 |
