# Supplementary material for: Metabolomics analysis identifies glutamic acid and cystine imbalances in COVID-19 patients without comorbid conditions. Implications on redox homeostasis and COVID-19 pathophysiology
Source: PLoS One. 2022 Sep 20;17(9):e0274910. doi: 10.1371/journal.pone.0274910 (PMC9488784; doi:10.1371/journal.pone.0274910)
Supplement: S2 Table — (DOCX) [file pone.0274910.s003.docx]

**Metabolomics Analysis Identifies Glutamic acid and Cystine imbalances in COVID-19 Patients Without Comorbid Conditions. Implications on Redox Homeostasis and COVID-19 Pathophysiology**

José C. Páez-Franco, José L. Maravillas-Montero, Nancy R. Mejía-Domínguez, Jiram Torres-Ruiz, Karla M. Tamez-Torres, Alfredo Pérez-Fragoso^3^ Juan Manuel Germán-Acacio^1^, Alfredo Ponce-de-León^4^, Diana Gómez-Martín^3^, and Alfredo Ulloa-Aguirre

**Supplementary information, S2 Table**

S2 Table. List of metabolites identified in the present GC/MS analysis

| # | Metabolites - ID | HMDB - ID | RT | %RSD | Kruskal Wallis – Dunn Test  P-value | | |
| --- | --- | --- | --- | --- | --- | --- | --- |
|  |  |  |  |  | HvsM | HvsS | MvsS |
| 1 | alpha-Hydroxybutyric acid | HMDB0000008 | 7.4621852 | 5.1892611 | >0.9999 | 0.0779 | 0.2271 |
| 2 | 3-Hydroxybutanoic acid | HMDB00011 | 7.8875455 | 6.4408233 | 0.2027 | 0.0355 | 0.4005 |
| 3 | alpha-Hydroxyisovaleric acid | HMDB00407 | 7.9712545 | 4.5381576 | >0.9999 | 0.019 | 0.032 |
| 4 | Aminobutyric acid | HMDB00687 | 8.0653091 | 11.609825 | >0.9999 | 0.0303 | 0.0582 |
| 5 | Valine 2TMS | HMDB0000883 | 8.6754545 | 7.8023825 | 0.335 | >0.9999 | >0.9999 |
| 6 | Leucine 2TMS | HMDB0000687 | 9.4366364 | 7.9970895 | >0.9999 | 0.0816 | 0.3382 |
| 7 | Glycerol | HMDB00131 | 9.4906182 | 6.2766516 | >0.9999 | 0.4838 | 0.8372 |
| 8 | Isoleucine 2TMS | HMDB0000172 | 9.7389091 | 6.0620644 | >0.9999 | >0.9999 | 0.6609 |
| 9 | Proline 2TMS | HMDB00162 | 9.7792364 | 9.1380725 | 0.1115 | 0.0006 | 0.0901 |
| 10 | Glyceric acid | HMDB00139 | 10.255655 | 6.7311948 | 0.0313 | 0.2953 | >0.9999 |
| 11 | Serine 3TMS | HMDB00187 | 10.646782 | 7.7826646 | <0.0001 | 0.1054 | 0.8604 |
| 12 | Threonine 3TMS | HMDB0000167 | 11.009618 | 6.7200068 | 0.8596 | >0.9999 | >0.9999 |
| 13 | Methionine 2TMS | HMDB0000696 | 12.634345 | 7.7737916 | >0.9999 | >0.9999 | >0.9999 |
| 14 | Pyroglutamic acid | HMDB0000267 | 12.676491 | 15.410306 | 0.0215 | >0.9999 | 0.0956 |
| 15 | 4-Hydroxyproline | HMDB0000725 | 12.754891 | 6.6824348 | 0.0286 | 0.3889 | >0.9999 |
| 16 | Threonic acid iso | HMDB00943 | 13.054055 | 5.9941382 | 0.4045 | >0.9999 | 0.124 |
| 17 | Threonic acid | HMDB00943 | 13.251082 | 8.184506 | 0.0747 | 0.0001 | 0.0481 |
| 18 | Glutamic acid 3TMS | HMDB00148 | 13.846782 | 7.2051406 | 0.0013 | <0.0001 | 0.0919 |
| 19 | Phenylalanine 2TMS | HMDB00159 | 13.929182 | 7.1341728 | 0.0237 | 0.0816 | >0.9999 |
| 20 | Lysine 3TMS | HMDB00182 | 14.83022 | 28.547765 | >0.9999 | >0.9999 | 0.507 |
| 21 | Glycerol phosphate | HMDB02520 | 15.549491 | 13.627297 | >0.9999 | 0.0159 | 0.0694 |
| 22 | Glutamine 3TMS | HMDB00641 | 15.572691 | 13.132458 | 0.2942 | 0.0002 | 0.0148 |
| 23 | Ornithine 4TMS | HMDB00214 | 16.070727 | 8.0276078 | 0.0879 | 0.1263 | >0.9999 |
| 24 | Citric acid | HMDB0000094 | 16.140164 | 12.685438 | 0.0953 | >0.9999 | 0.2168 |
| 25 | 1,5-Anhydroglucitol | HMDB0002712 | 16.491327 | 5.7009337 | >0.9999 | 0.1886 | 0.1886 |
| 26 | Tyrosine 3TMS | HMDB00158 | 17.319673 | 7.3110517 | 0.9956 | 0.0876 | 0.4291 |
| 27 | N-acetyl-L-lysine | HMDB00206 | 17.375309 | 6.14023 | 0.8813 | >0.9999 | 0.5263 |
| 28 | Myo-inositol | HMDB0000211 | 18.918582 | 10.02933 | >0.9999 | 0.6347 | >0.9999 |
| 29 | Uric acid | HMDB00289 | 18.938745 | 13.323636 | 0.0103 | 0.0246 | >0.9999 |
| 30 | Linoleic acid | HMDB00673 | 19.691818 | 8.134679 | >0.9999 | 0.4934 | 0.2905 |
| 31 | Palmitoleic acid | HMDB0003229 | 19.742055 | 4.0026969 | >0.9999 | 0.0374 | 0.2076 |
| 32 | Cystine | HMDB00192 | 20.6158 | 16.887837 | 0.0196 | <0.0001 | 0.0928 |
| 33 | Cholesterol | HMDB00067 | 26.890364 | 7.4239132 | 0.0248 | 0.0008 | 0.2654 |
